# Complete plastome sequence of *Elaeagnus glabra* (Elaeagnaceae): an Asian endemic plant species

**DOI:** 10.1080/23802359.2019.1702483

**Published:** 2019-12-13

**Authors:** Kun-Kun Zhao, Jian-Hua Wang, Zhi-Xin Zhu, Guo-Zheng Shi, Shui-Xing Luo, Hua-Feng Wang

**Affiliations:** a The Experimental Station of Research Institute of Tropical Forestry, Chinese Academy of Forestry, Jianfeng, China;; b Key Laboratory of Tropical Biological Resources of Ministry of Education, School of Life and Pharmaceutical Sciences, Hainan University, Haikou, China

**Keywords:** *Elaeagnus glabra*, complete plastome, phylogeny, genome structure, Rosales

## Abstract

*Elaeagnus glabra* is an evergreen vine or climbing shrub with 5 m height. It is widespread in southern China. It grows in the sunny forests or forest margins below 1000 m a.s.l. In this paper, we report and describe the complete plastome of *E. glabra* in order to provide useful genomic data for its systematic research. The complete plastome of *E. glabra* is 152,555 bp with a typical quadripartite structure of angiosperms. It contains two Inverted Repeats (IRs) of 25,918 bp, a large single-copy (LSC) of 82,408 bp, and a small single-copy (SSC) region of 18,311 bp. The complete plastome contains 129 genes, including 83 protein-coding genes, 38 tRNA genes, and eight rRNA genes. The overall A/T content in the chloroplast genome of *E. glabra* is 62.90%. The phylogenetic analysis indicated that *E. glabra* is close to *E. loureirii* within Elaeagnaceae. The complete plastome of *E. glabra* will provide useful resources for the development and utilization of this species and the phylogenetic study of Rosales.

## Introduction

*Elaeagnus glabra* is an evergreen vine or climbing shrub with 5 m height. It is widespread in southern China, including Jiangsu, Zhejiang, Fujian, Taiwan, Anhui, Jiangxi, Hubei, Hunan, Sichuan, Guizhou, Guangdong, and Guangxi, as well as Japan and it often grows in sunny forests or forest margin below 1000 m a.s.l. (Sun and Lin 2010). Fruits can be eaten, leaves can be used medicinally, stem bark can be used as paper or man-made fibers (Zhu et al. 2017). It has high economic value and scientific research value. Here, we report and characterize the complete plastome of *E. glabra* (GenBank accession number: MN306571). This is the first report of a complete plastome for *E. glabra*.

The *E. glabra* samples in this study were taken from Ulleungdo island, south Korea (N37.483°, E130.9°). A voucher specimen and its DNA (Chung et al., eg1) was deposited in the Herbarium of the Institute of Tropical Agriculture and Forestry (HUTB), Hainan University, Haikou, China. We employed the modified CTAB method (Doyle and Doyle 1987) to extract the total genomic DNA of *E. glabra* from silica gel-dried leaves.

Whole-genome short-gun sequencing was performed on the Illumina Hiseq 2500 platform, with the 150 bp paired-end sequencing method. We carried out quality control of sequenced genomic data and cleaning up unqualified sequences (Patel and Jain 2012). Finally, about six GB of clean data is obtained. Using *E macrophylla* plastome sequence (KP211788.1) (Choi et al. 2015) as template, the plastome of *E. glabra* was assembled by MITObim v1.8 (Hahn et al. 2013). The completeplastome was annotated using Geneious R8.0.2 (Biomatters Ltd., Auckland, New Zealand) against the chloroplast genome of *E. macrophylla* (KP211788.1). The annotation was corrected with DOGMA (Wyman et al. 2004).

The complete plastome of *E. glabra* was found to be 152,555 bp in length with the typical quadripartite structure of angiosperms, contains two Inverted Repeats (IRs) of 25,918 bp, a large single copy (LSC) region of 82,408 bp, a small single-copy (SSC) region of 18,311 bp. The complete plastome contains 129 genes, including 83 protein-coding genes, 38 tRNA genes, and eight rRNA genes. The overall A/T content in the chloroplast genome of *E. glabra* is 62.90%, in which the corresponding values of the LSC, SSC, and IR region were 65.00%, 69.50%, and 57.20%, respectively.

**Figure 1.**
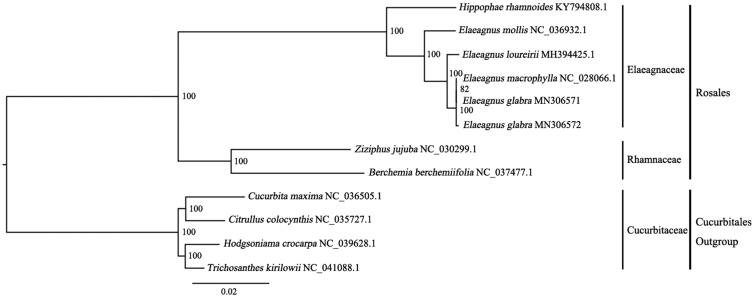
Phylogenetic tree (ML) based on eight complete plastome sequences of Rosales. Accession numbers: *Berchemia berchemiifolia* NC_037477.1, *Ziziphus jujuba* NC_030299.1, *Hippophae rhamnoides* KY794808.1, *Elaeagnus mollis* NC_036932.1, *E loureirii* MH394425.1, *E glabra* MN306571 (this study), *E glabra* MN306572, *E macrophylla* NC_028066.1; Outgroups: *Cucurbita maxima* NC_036505.1, *Citrullus colocynthis* NC_035727.1, *Hodgsonia macrocarpa* NC_039628.1, and *Trichosanthes kirilowii* NC_041088.1.

A maximum-likelihood (ML) phylogenetic tree of the eight published complete plastomes of Rosales (plus *E. glabra*) was built with RAxML (Stamatakis 2006), using *Cucurbita maxima*, *Citrullus colocynthis*, *Hodgsonia crocarpa*, and *Trichosanthes kirilowii* as outgroups (Figure 1). The phylogenetic analysis indicated that all members of Rosales were clustered with a high bootstrap support value and there was a close relationship between *E. glabra* and *E. macrophylla*. In this study, we report the characterization of the complete plastomes of *E. glabra* for the first time, which may provide a useful resource for the development and utilization of *E. glabra*, and also for phylogenetic studies of *E. glabra*.
